# Infectious Disease Does Not Impact the Lying and Grooming Behaviour of Post-Parturient Dairy Cows

**DOI:** 10.3390/ani9090634

**Published:** 2019-08-30

**Authors:** Nadège Perier, Alice de Boyer des Roches, Margit Bak Jensen, Kathryn Proudfoot

**Affiliations:** 1 Université de Lyon, VetAgro Sup, Marcy L’Etoile F-69280, France; 2 Université Clermont, Auvergne, INRA, VetAgro Sup, UMR Herbivores, Saint-Genès-Champanelle 63122, France; 3 Department of Animal Science, Aarhus University, Blichers Allé 20, Tjele DK-8830, Denmark; 4 Veterinary Preventive Medicine, College of Veterinary Medicine, The Ohio State University, 1920 Coffey Road, Columbus, OH 43210, USA

**Keywords:** maternal behaviour, sickness behaviour, dairy cow, motivation, health status

## Abstract

**Simple Summary:**

Animals reduce their activity, feeding behaviour, social interactions and self-grooming behaviours when sick (‘sickness behaviours’). However, the effect of sickness on early maternal behaviours directed toward offspring is less understood, especially in farm animals. This experiment assessed the impact of sickness on the first day after giving birth on the lying and grooming behaviour of dairy cows and their calves. The behaviours of sick and healthy cows and their calves were recorded for 24 hours after calving. Behaviours included the lying behaviour of the cow and calf, and maternal grooming of the calf. We found that health status did not affect the majority of the behaviours measured after calving. We suggest that a cow’s motivation to groom and be near their calf may be stronger than her motivation to express sickness behaviours immediately after giving birth.

**Abstract:**

Behaviour is commonly used to detect sickness in animals, but the impact of sickness on lying and maternal behaviours around parturition is not well understood. The objective was to assess the effects of sickness on the lying and grooming behaviours of dairy cows in the first 24 h after giving birth. Cows were categorized as ‘sick’ (n = 8) if they had at least one rectal temperature ≥39.1 °C and one clinical sign of illness (mastitis, pneumonia or an unknown infection) within 24 h of calving. These cows were match-paired for parity with cows that had no rectal temperature ≥39.1 °C and no clinical signs of illness up to 3 d after calving (n = 8; ‘not sick’). The duration and latency of cow behaviours (standing, lying, lying bouts, lying close to calf, and grooming of the calf) and calf behaviours (standing and lying) were recorded for 24 h post-partum. We found no differences in the behaviour of sick and not sick cows and their calves post-calving, except that sick cows took longer to lie down near their calf after calving compared to those without illness. Cows may be more motivated to groom and spend time with their calf than to express sickness behaviours immediately after giving birth.

## 1. Introduction

The majority of dairy cow diseases appear during the month following calving [1,2]. Early disease detection is important to ensure that cows are treated promptly. Behaviour can be used by farmers and veterinarians to identify sick animals [3]; sick animals reduce their activity, feeding, social interactions and hedonic behaviours [4]. These behavioural changes have been described in dairy cows with mastitis [5,6,7,8]; metritis [9,10,11]; and lameness [12,13,14]. However, no study has explored the effect of infectious diseases on cows’ behaviour in the few hours immediately following parturition, including on maternal behaviours directed toward the calf.

In sheep and beef cattle production systems where offspring are raised with their dam or ewe until weaning, maternal behaviour is essential for the survival of the young [15,16]. In dairy cattle, the dam and calf are often separated within a few hours of birth, however, new housing systems that incorporate more care of the calf from the dam are being explored [17]. When the dam and calf are kept together after birth, the dam dedicates the majority of her time standing and grooming the calf in the first 6 h, after which she spends more time lying down and feeding [18]. This early grooming behaviour stimulates the calf’s activity, respiration, blood circulation as well as urination and defecation [19]. The dam also spends the majority of her time near the calf in the first 6 h after calving, likely to protect her newborn from danger [20].

The influence of early disease in dairy cows on lying and grooming behaviours immediately after calving is not well understood. Thus, the objective was to determine the effect of sickness on the lying behaviour of the cow and her calf, as well as the maternal grooming behaviour of the cow directed toward the calf.

## 2. Materials and Methods

The study was conducted from September 2011 to February 2012 at the Aarhus University cattle research facility (Foulum, Denmark) and in accordance with a protocol approved by the Danish Animal Experiments Inspectorate (Journal no 2010/561−1780).

### 2.1. Animals and Housing

The experiment began with 79 multiparous Danish Holstein dairy cows that were included in a larger study assessing the impact of moving cows into maternity pens at different time periods relative to calving [21]. Before calving, cows were grouped into 1 of 6 blocks of 14.7 ± 1.5 (mean ± SD) cows based on expected calving date. Approximatively two weeks before their expected calving date, cows were moved into group pens (9 × 15 m) with deep straw bedding. Cows were fed a total mixed ration (TMR) ad libitum at 1000 and 1700 h with a forage-to-concentrate ratio of 79:21 (%, DM basis) before calving and 60:40 (%, DM basis) after calving.

Either before or during signs of calving, cows were moved into individual maternity pens (each 3.0 × 4.5 m with deep-bedded straw). The time at which cows were moved into the individual pen was dependent on treatments assigned to them as part of the larger study [21]. After calving, the cow and her calf remained in the individual pen for 3 d. As part of the larger study, approximately half of the individual pens were ‘partially covered’ in plywood to assess the impact of a secluded area on calving location [22].

In the individual pens, cows were milked twice daily at 06:00 and 18:00 using a manual milking machine. Water was available ad libitum in all pens with drinking bowls. All calves were offered 4 L of colostrum from a bottle within 6 h of birth. If the calf consumed less than 3.5 L, it was offered the remaining amount 2 h later, unless the staff assessed that the calf had been suckling the dam. Farm staff monitored the calves and assisted with suckling or bottle-fed them when they appeared to need assistance. On the third day after calving, the cow and calf were separated; the cow was moved into a free-stall pen with other lactating cows and the calf was moved to the nursery.

### 2.2. Inclusion Criteria and Health Status

To determine if cows were sick after calving, data were collected from veterinary records, daily rectal temperatures and records of abnormal milk from the milker during the first 3 d after calving. The herd veterinarian visited the farm as needed when farm staff recognized signs of illness in a cow (mastitis, pneumonia or an unknown infection). Rectal body temperatures were taken by the milker twice daily for the first 3 days after calving. Signs of mastitis were recorded twice daily by the milker based on the colour and consistency of the milk. Using these data, we defined ‘sick’ cows as those with at least one high rectal body temperature (≥39.1 °C) and one clinical sign of illness diagnosed by the veterinarian and/or milker within 24 h post calving.

These sick cows were then match-paired with cows that had no rectal temperature ≥39.1 °C and no clinical signs of illness for 3 d post calving (‘not sick’). We determined if cows were sick or not on d 3 after calving using a clinical health examination. The clinical examination included a vaginal discharge score to diagnose acute metritis (4-point system; see [9] for a description), and a blood sample taken from the tail vein to diagnose clinical and subclinical ketosis (Precision Xtra Ketone Glucose and Ketone Monitoring System, Abbott Laboratories, Chicago, IL; validated by [23]). Sick and not sick cows were matched for parity, time spent in the maternity pen, and whether they were in the partially covered pen or in the non-covered pen of the earlier study [21,22].

When a cow was diagnosed with mastitis, pneumonia or an infection, she was treated on the day of diagnosis with an injectable antibiotic according to standard veterinary procedures—pneumonia: Alamycin 300 mg/mL, 100 mL dosage (Norbrook Laboratories Ltd., Corby, UK) or Aquacycline vet 100 mg/mL, 100 mL dosage (Ceva Santé Animale, Libourne, France; mastitis: penicillin, 100 mL dosage (Penovet; Boehringer Ingelheim Pharma GmbH and Co. KG, Ingelheim, Germany); amoxicillin, 150 mL dosage (150 mg/mL Curamox; Boehringer Ingelheim Denmark A/S, Copenhagen, Denmark); or sulfadiazine 200 mg and trimethoprim 40 mg, 150 mL dosage (Norodine 24%; Norbrook Laboratories Ltd., Newry, United Kingdom), steptomycin and penicillin G, 200 mg/mL, 100 mL dosage (STREPTICILLIN VET, Intracin Pharmaceutical Pvt. Ltd., Gujarat, India), cloxacillin 200mg and ampicillin 75 mg (LACTACLOX VET, Norbrook Laboratories Ltd., Newry, Northern Ireland) or cefoperazone 250 mg (PATHOZONE, Zoetis, Malakoff, France); infection: Alamycin 300 mg/mL, 100 mL dosage (Norbrook Laboratories Ltd., Corby, UK), Aquacycline vet 100 mg/mL, 100mL dosage (Ceva Santé Animale, Libourne, or penicillin, 100 mL dosage (Penovet; Boehringer Ingelheim Pharma GmbH and Co. KG, Ingelheim, Germany). Treatments were repeated by farm staff for 3 d after diagnosis. Cows were also given a nonsteroidal anti-inflammatory drug (NSAID) on the day after diagnosis (either flunixin meglumine, 50 mg/mL, 20 mL dosage (Finadyne Solution; MSD Animal Health, Milton Keynes, UK) or Metacam, 20 mg/mL solution, 15 mL dosage (Boehringer Ingelheim Pharma GmbH and Co. KG)) with or without a steroidal anti-inflammatory drug (SAID) (dexamethasone, 2 mg/mL, FLUORMETHYLPREDNISOLON^®^, ScanVet Animal Health A/S, Fredensborg, Denmark).

Cows were not included in the experiment if they had calved in the group pen before being moved into the individual pen, experienced a difficult calving (dystocia; recorded as any calving assistance from farm staff), had twins, or were diagnosed with milk fever in the first day after calving.

### 2.3. Behavioural Data Collection

The behaviour of cows and their calves was recorded for 24 h post-partum using a digital video camera (TVCCD-140IR; Monacor UK Ltd., Milton Keynes, UK) mounted above each individual pen. The moment of calving was recorded for each cow and was defined as the time when the calf’s hips were fully expelled from the cow. Table 1 describes behaviours collected in this study; all behaviours were continuously collected from video.

The start and end time for each behaviour was recorded using Microsoft Excel during the 24 h after birth. Cow and calf lying bouts were calculated as the number of times animals transitioned from standing to lying. Raw data were used to calculate the latency of each behaviour from the moment of calving (min) and the duration of each bout (min/bout). The latency for the calf to lie down was considered the first time the calf laid down after standing for the first time.

### 2.4. Statistical Analysis

The cow and calf dyad was considered the experimental unit. Before analysis, behavioural data were summarized into the period 24 h after calving, as well as a sum for each 6 h period after calving (1 to 6 h, 7 to 12 h, 13 to 18 h and 19 to 24 h).

All statistical analyses were performed using SAS software (version 9.4; SAS Institute Inc., Cary, NC, country). The normality of residuals and of random effect distribution were visually assessed using plots of residuals and quantile–quantile plots of residuals and random effects. Significance was declared at *p* < 0.05. We anticipated that parity would impact the behaviours based on previous literature [18], thus, parity was forced into all models as a categorical variable (2 and 3 vs. 4).

To determine the impact of sickness on the daily duration of cow-calf behaviours, a linear mixed model (PROC MIXED) was used. The model included parity (2 and 3 vs. 4) and health status (sick or not sick) as fixed effects, a parity by health status interaction, and a matched pair as a random effect. Non-significant interactions were removed from models using backward manual elimination.

To determine the impact of sickness on the duration of cow-calf behaviours across 6 h periods, a linear mixed model was used (PROC MIXED). The model included parity (2 and 3 vs. 4) and health status (sick or not sick) as fixed effects, a 6 h period as a repeated measure, all interactions between health, parity and period, and a matched pair as a random effect. The cow-calf pair was considered the subject of the repeated measure, and an autoregressive covariance structure was chosen based on best fit. Non-significant interactions were removed from models using backward manual elimination. Based on a visual inspection of the graphs, we conducted post-hoc contrasts between the first period (1 to 6 h after calving) and the remaining periods averaged together for all behaviours using the ESTIMATE statement.

A cox proportional hazards regression model (PROC PHREG) was used to determine the impact of health status (sick or not sick) and parity (2 and 3 vs. 4) on the latencies of behaviours (cow lying, cow standing, calf lying, calf standing, cow-calf lying close and initiation of grooming) after calving. The MEANS procedure was used to generate descriptive means and standard errors for latency behaviours.

## 3. Results

### 3.1. Animals Included in the Study

From the 79 cows initially included in the study, 10 were excluded because they calved in the group pen, 7 were excluded due to dystocia or delivering twins and 2 were excluded due to milk fever. Of the remaining 60 cows, 8 met our criteria for ‘sick’ cows (mastitis n = 3, pneumonia n = 1, unknown infection n = 4) and 30 met our criteria for ‘not sick’. Twenty-two cows were excluded due to not meeting our inclusion criteria (e.g., fever but not clinical signs of illness, or clinical sign of illness >24 h and <3 d after calving). Of the 30 ‘not sick’ cows, 8 were match-paired with the sick cows based on parity (mean ± SD; sick: 2.25 ± 0.9, not sick: 2.25 ± 0.9; parity as a categorical variable: 2 and 3 vs. 4; sick: 4 vs. 4, not sick: 4 vs. 4), time in the maternity pen before calving (mean ± SD; sick: 87.6 ± 115.7 not sick: 33.3 ± 39.0 min), and inclusion in the previous study with a partially covered area of the pen (sick: 5, not sick: 5).

### 3.2. Behaviour of the Cow and Calf in the 24 h after Calving

There was no effect of sickness on the daily duration of grooming behaviour directed toward the calf (sick vs. not sick: 2.4 vs. 2.6 ± 0.2 h/24 h; *p* = 0.41).

There was no effect of sickness on the duration of daily lying time for the cow (sick vs. not sick: 12.7 vs. 12.1 ± 0.7 h/24 h; *p* = 0.37) or her calf (sick vs. not sick: 18.7 vs. 18.8 ± 0.5 h/24 h; *p* = 0.93). There was no effect of sickness on lying bouts of the cow (sick vs. not sick: 25 vs. 25 ± 2 n/24 h; *p* = 0.83) or her calf (sick vs. not sick: 34 vs. 30 ± 3 n/24 h; *p* = 0.30). There was also no effect of sickness on the duration of time that the dam and calf spent lying close to each other (sick vs. not sick: 10.2 vs. 10.0 ± 0.8 h/24 h; *p* = 0.87).

There was no effect of parity on any behavioural measurements in the 24 h after calving.

### 3.3. Behaviour of the Cow and Calf over 6 h Periods

There was no effect of sickness on the latency for the cow to start grooming her calf, for the cow to stand or lie down, or for the calf to stand or lie down (Table 2). There was an effect of parity (*p* = 0.04) and health status (*p* = 0.04) for latency for the cow-calf pair to lie down close together for the first time after birth. Sick cow-calf pairs (Table 1), and those with dams of lower parity (2 and 3 vs. 4: 85.2 ± 14.1 vs. 52.6 ± 9.7 min) had a longer latency to lie down close together compared to those that were not sick and of higher parity.

**Figure 1:**
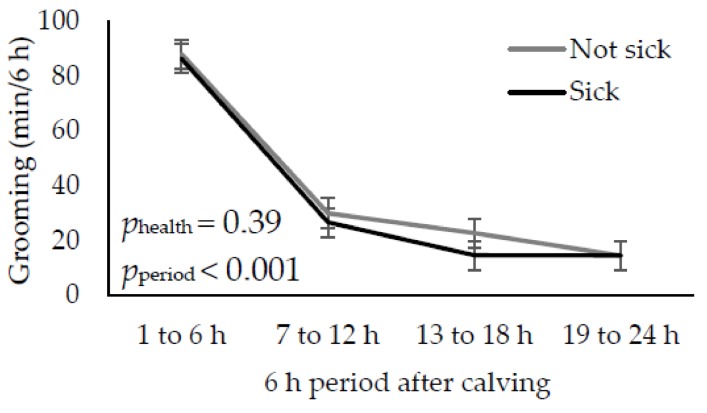
Least square means and standard errors for grooming behaviour directed toward the calf in the 24 h after calving for cows that were ‘sick’ (clinical sign of illness and rectal temperature ≥39.1 °C 9 within 24 h after calving) and ‘not sick’ (no clinical sign of illness and rectal temperature ≥39.1 °C within 3 d after calving) across 6 h periods.

There was no effect of health status on grooming behaviour across 6 h periods (Figure 1). There was an effect of period (*p* < 0.001), whereby cows spent the most time grooming their calves in the 6 h after calving compared to later periods (*p* < 0.001).

**Figure 2:**
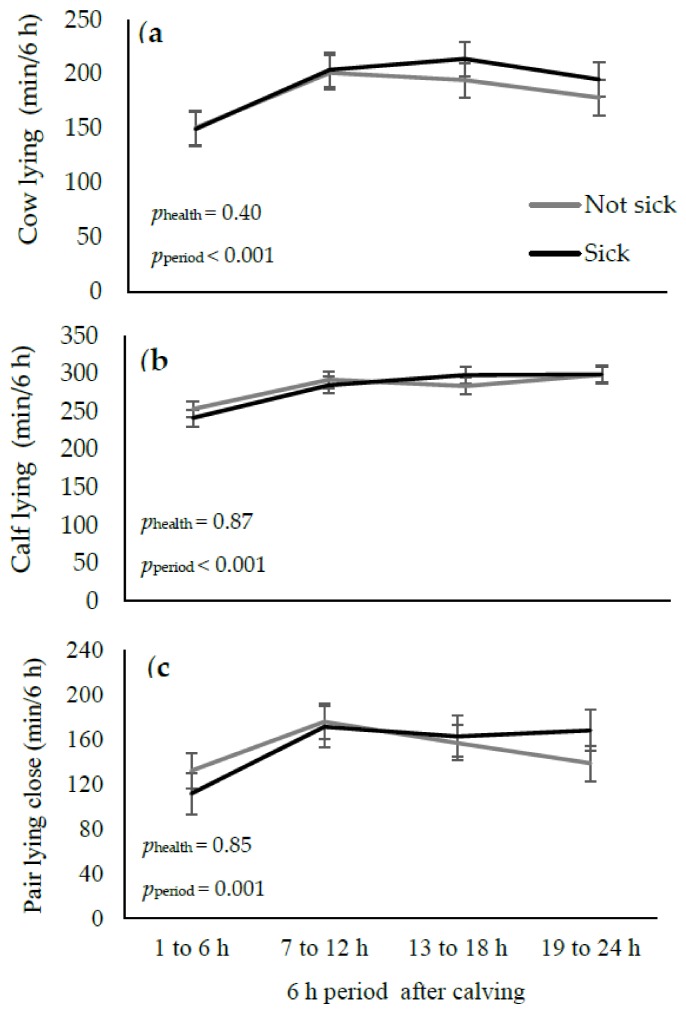
LS means and SE for lying behaviour of ‘sick’ (black lines; clinical sign of illness and rectal temperature ≥39.1 °C 9 within 24 h after calving) and ‘not sick’ (no clinical sign of illness and rectal temperature ≥39.1 °C within 3 d after calving) cows and their calves in the 24 h after calving across 6 h periods: (**a**) Lying time of the cow; (**b**) Lying time of the calf; (**c**) Time that the cow-calf pair spent lying close together (within ½ calf length).

There was no effect of health status on the duration that the cow and her calf spent lying, nor on the duration of time the cow-calf pair spent lying close together (Figure 2). Regardless of health status, the lying time of the cow (*p* < 0.001) and calf (*p* < 0.001), as well as the time that the pair spent lying close to each other (*p* = 0.002) was lowest in the 6 h after calving compared to later periods (Figure 2). There was no effect of parity on any lying behaviour of the cow and calf.

The number of lying bouts made by the cow and her calf was not affected by sickness across 6 h periods (cow sick vs. not sick: 6 vs. 6 ± 1 n/6 h; *p* = 0.83; calf sick vs. not sick: 9 vs. 8 ± 1; *p* = 0.22). Regardless of health status, there was an effect of the 6 h period on lying bouts (*p* < 0.001 for both); both cows (*p* < 0.001) and their calves (*p* < 0.001) had higher lying bouts in the 6 h period after calving compared to later periods.

## 4. Discussion

The objectives of the study were to determine the effect of sickness on the lying behaviour of the cow and her calf, as well as maternal grooming behaviour of the cow directed toward the calf. Overall, we detected no major differences in the duration and latencies of these behaviours, except that sick cows took longer to lie down close to their calves compared to those that were not ill. These findings suggest that cows were more motivated to care for their calf than to express classic sickness behaviours, such as an increased lying time and reduced social behaviours immediately after giving birth.

There was no effect of sickness on the duration of time the dam spent grooming her calf in the first 24 h after birth. This result suggests that grooming offspring immediately after birth may not be reduced during illness as other social behaviours are [24,25]. Although no research has assessed the impact of sickness on maternal grooming of offspring, cows with mastitis [5] and metritis [26] reduce self-grooming behaviour when ill. Mandel et al. [26] suggest that self-grooming is a ‘low-resilience’ behaviour that cows give up when energy resources are low, such as during illness. However, maternal grooming after birth was not given up in our study and dams were likely strongly motivated to perform behaviours that ensure the survival of their offspring soon after birth. Maternal grooming has been found to encourage the development of the dam-calf bond, as well as to stimulate the calf’s activity, respiration, blood circulation as well as urination and defecation, thus increasing the calf’s likelihood of survival [19]. Although not studied in dairy cows, research using mice has found that dams maintain some maternal behaviours (nest-building and pup-retrieval) when experimentally induced with illness using LPS [27]. These maternal behaviours were the strongest when thermal conditions put the lives of their pups at risk (6 °C compared to 22 °C). Like the mice, the cows in our study may have prioritized the maternal grooming of their calves over expressing sickness behaviours such as lethargy and asocial behaviour.

Regardless of health status, cows groomed their calves the most in the first 6 h after birth compared to later periods. These results are in accordance with previous studies [18,20,28,29]. Regardless of health status, cows began to groom their calf about 4 min after calving. This result is similar to previous work, where latencies for grooming have been reported as between 42 s and 3 min [18,28,29].

Regardless of health status, cows in our study stood up about 4 min after calving and laid down for the first time about 58 min after calving. The latency to stand reported here is slightly late compared to latencies described in the literature (i.e., 30 s to 3.7 min) [18,20,21,22,23,24,25,26,27,28,29]. Sick cows laid down close to their calf 32 min later than healthy cows. This result suggested that sick cows may have delayed some facets of their maternal behaviour. It is not clear whether the cow or her calf were the cause of this delayed latency to lie down close together. However, we speculate that sick cows may have delayed lying down close to their calf due to lethargy associated with the illness [25], as sick cows may have been less willing to move with their calf until the calf settled into a lying position.

There was no effect of sickness on the duration of time that the dam-calf pair spent lying close together. Like grooming, this behaviour may be prioritized by the cow to help ensure the survival of her young. Similarly, Edwards and Broom [20] reported that cows stayed within 1 m of their calf for the six hours after parturition (standing or lying). The proximity of the dam and her calf may be affected by the space and resources allotted to them. For example, in feral cattle, the dam stays near the calf for the first few hours after birth, but then leaves the calf in a secluded area to hide for the first few days after birth while she grazes nearby [30]. In a study of indoor-housed cows kept in large group pens with access to secluded areas, researchers found that cow-calf pairs will move into secluded areas in the few hours after birth [31]. In our case, the pen size was small (3.0 × 4.5 m), so cow-calf pairs may have been limited in their ability to move greater distances than if they were provided more space and access to secluded areas.

We found no impact of sickness on lying time and lying bouts of the dam or her calf. An increase in lying time is typical when mammals become ill with an infectious disease, as the immune system requires conservation of energy to help the animal recover [24,25]. This lack of difference may be driven in part by the variability of diseases included in the sick group. For example, multiple studies have found that cows with mastitis spend more time standing when ill, perhaps because lying puts pressure on their udder, causing pain [5,6,32,33]. Indeed, cows with mastitis will avoid lying down in the side with the infected quarter [32]. In our study, three of the eight sick cows had mastitis, which may have interfered with our ability to detect an increase in lying time due to sickness. Another reason for this lack of difference may be the strong motivation of the cow to stand and lick or nurse her calf in the few hours after calving, prioritizing these behaviours over resting.

Despite no impact of sickness on lying time or lying bouts, all cows had lower lying durations and a higher number of lying bouts in the 6 h after calving compared to later periods. These findings, similarly to grooming behaviour, reflect intensive maternal behaviour and interaction between the dam and calf during the initial hours after calving [18].

There was no effect of the dam’s health status on any behaviour of the calf. This finding is not surprising given that health status did not impact the behaviour of the dam. Calves appeared to be most active in the first 6 h after calving compared to later periods, which is consistent with another study that measured the behaviour of calves kept with their dams for the first 3 d after birth [18]. Calves stood up for the first time at approximately 74 min after calving; this result is in the same range (32 to 105 min) as previous studies [18,20]. More research is needed to determine any detrimental impacts of the dam’s health status on nursing/suckling between the pair, as well as any long-term effects of illness on the ability of the dam to care for her calf.

The main limitations of the study are the small sample size, the variability in diseases included in the sick group (mastitis, pneumonia and an unknown infection), and the inability to measure nursing and suckling behaviour between the dam-calf pair. We were not able to accurately measure nursing/suckling behaviour, as the farm staff would occasionally help the calf nurse the dam in the first 24 h of life, and would at other occasions provide the calf with milk from a bottle. This interaction between farm staff and calves did not appear to be driven by the health status of the cow, and we were not able to control for these interactions, so these behaviours were not included in the study. Future studies are encouraged to determine the impact of specific diseases on behaviour and nursing/sucking behaviour should be included as part of the data collection. It is also unclear whether the presence of the calf specifically impacted sickness behaviour in this study, as we were not able to compare the cows’ behaviour with and without their calves. Future work is recommended to determine if the presence of the calf is a main driver in the lack of response reported in this study.

## 5. Conclusions

Our study showed that health status did not affect the lying and grooming behaviour of a cow-calf pair. However, sick cows laid down close to their calf 32 min later than healthy cows, suggesting that sick cows may have delayed some facets of their maternal behaviour. Cows seemed to be more motivated to express maternal behaviours that may increase the likelihood of calf survival (grooming and lying close) than expressing classic sickness behaviours (increased lying behaviour and decreased social behaviour) in the first 24 h after calving. These results suggest that behaviour may not be a useful tool for detecting sickness in dairy cows immediately after calving, although more research using a larger sample of cows with each illness is still required.

## Figures and Tables

**Table 1:** Definition of behaviours recorded in the 24 h after calving for ‘sick’ (clinical sign of illness and rectal temperature ≥39.1 °C 9 within 24 h after calving) and ‘not sick’ (no clinical sign of illness or rectal temperature ≥39.1 °C within 3 d after calving) dairy cows and their calves.

Behaviour	Definition
Cow standing upright	Cow is standing with her body supported by her four legs, or walking
Cow lying	Cow is lying on her sternum or side
Cow-calf lying close	Cow and calf are lying on sternum or on their side. Any part of the cow is positioned within ½ of a calf’s length from the calf
Cow grooming	Cow’s muzzle is in contact with, or in close proximity to, any part of the calf’s body
Calf standing upright	Calf is standing with all four legs fully extended for longer than 5 s, or is walking
Calf lying	Calf is lying on sternum or side

**Table 2:** Means and standard errors for the latency of ‘sick’ (clinical sign of illness and rectal temperature ≥39.1 °C 9 within 24 h after calving) and ‘not sick’ (no clinical sign of illness and rectal temperature ≥39.1 °C within 3 d after calving) cows and their calves to perform behaviours after the moment of calving.

Latency	Health Status				*p* > ChiSq	Hazard Ratio
	Not Sick	SE^1^	Sick	SE^1^		
Cow latency to groom calf	4.5	1.3	3.0	2.2	0.16	0.46
Cow latency to stand	4.4	1.3	3.0	2.2	0.20	0.48
Cow latency to lie down	52.7	4.8	63.5	11.5	0.21	2.03
Calf latency to stand	86.9	17.5	60.3	11.5	0.28	0.56
Calf latency to lie down	96.0	15.7	64.2	11.9	0.15	0.43
Cow-calf latency to lie close together	52.7	4.8	85.1	16.5	0.04	4.00

^1^ Standard error.
